# Characterization of the upper respiratory tract microbiota in Chilean asthmatic children reveals compositional, functional, and structural differences

**DOI:** 10.3389/falgy.2023.1223306

**Published:** 2023-07-28

**Authors:** Ignacio Ramos-Tapia, Katiuska L. Reynaldos-Grandón, Marcos Pérez-Losada, Eduardo Castro-Nallar

**Affiliations:** ^1^ Centro de Bioinformática y Biología Integrativa, Facultad de Ciencias de la Vida, Universidad Andrés Bello, Santiago, Chile; ^2^ Facultad de Enfermería, Universidad Andrés Bello, Santiago, Chile; ^3^ Computational Biology Institute, Department of Biostatistics and Bioinformatics, The George Washington University, Washington, DC, United States; ^4^ Departamento de Microbiología, Facultad de Ciencias de la Salud, Universidad de Talca, Talca, Chile; ^5^ Centro de Ecología Integrativa, Universidad de Talca, Talca, Chile

**Keywords:** 16S rRNA, asthma, nasal bacteriome, Chilean microbiota, upper respiratory tract

## Abstract

Around 155 million people worldwide suffer from asthma. In Chile, the prevalence of this disease in children is around 15% and has a high impact in the health system. Studies suggest that asthma is caused by multiple factors, including host genetics, antibiotic use, and the development of the airway microbiota. Here, we used 16S rRNA high-throughput sequencing to characterize the nasal and oral mucosae of 63 asthmatic and 89 healthy children (152 samples) from Santiago, Chile. We found that the nasal mucosa was dominated by a high abundance of *Moraxella, Dolosigranulum, Haemophilus, Corynebacterium, Streptococcus,* and *Staphylococcus*. In turn, the oral mucosa was characterized by a high abundance of *Streptococcus*, *Haemophilus, Gemella, Veillonella, Neisseria*, and *Porphyromonas.* Our results showed significantly (*P* < 0.001) lower alpha diversity and an over-abundance of *Streptococcus* (*P* < 0.01) in nasal samples from asthmatics compared to samples from healthy subjects. Community structure, as revealed by co-occurrence networks, showed different microbial interactions in asthmatic and healthy subjects, particularly in the nasal microbiota. The networks revealed keystone genera in each body site, including *Prevotella, Leptotrichia,* and *Porphyromonas* in the nasal microbiota, and *Streptococcus, Granulicatella,* and *Veillonella* in the oral microbiota. We also detected 51 functional pathways differentially abundant on the nasal mucosa of asthmatic subjects, although only 13 pathways were overrepresented in the asthmatic subjects (*P* < 0.05). We did not find any significant differences in microbial taxonomic (composition and structure) and functional diversity between the oral mucosa of asthmatic and healthy subjects. This study explores for the first time the relationships between the upper respiratory airways bacteriome and asthma in Chile. It demonstrates that the nasal cavity of children from Santiago harbors unique bacterial communities and identifies potential taxonomic and functional biomarkers of pediatric asthma.

## Introduction

The World Health Organization estimates that around 250,000 people die each year globally due to asthma and that its prevalence will increase to 100 million people by 2025 (1). Asthma affects all age ranges, but it is a common chronic disease in children and adolescents (2–4); being their most common chronic respiratory condition worldwide, with around 14% of children and young people affected (4). In Chile, the prevalence of pediatric asthma is 15.5% (5, 6) and has a high economic impact on the health system with approximately 15 million dollars spent in treatment per year (7).

Many factors are involved in the onset and incidence of asthma, including host genetics (8), ethnic components (9), degrees of urbanization (10), gender (11), and environmental factors, e.g., dust exposure (12), living in a farm-like environment (13), antibiotic use during infancy (14), and mode of delivery (15). All of the aforementioned factors also contribute to the microbiota establishment, where an imbalance of the microbiota can lead to disease (16–19).

Several studies have described a relationship between the airway microbiota and the development of asthma, the vast majority revealing an imbalance or dysbiosis (17, 20–23). Some of those studies have also highlighted that the most predominant bacterial genera in the respiratory tract of asthmatic patients are *Moraxella*, *Haemophilus*, and *Streptococcus*, with *Moraxella catarrhalis*, *Haemophilus influenzae*, and Streptococcus *pneumoniae* being dominant in children with asthma (20, 24–28). While many of those studies have focused on the dysbiosis of healthy subjects compared to asthmatics and concurred that this dysbiosis is related to *Moraxella*, *Haemophilus*, and *Streptococcus*, fewer studies have explored changes in microbial interactions (29, 30).

The nostrils are a repository and point of entry of multiple pathogens to the lower respiratory tract (31). However, the oral cavity is the initial interface between allergens, microbiome, and mucosal immunity. The anatomical connection between the oral cavity and the lungs provides many opportunities for the oral microbiota to affect the lung microbiota in different situations (32–34). In fact, recent studies have found differences in the dental microbiota of subjects with asthma and atopy compared to healthy subjects (35). However, studies are still limited and often include few subjects.

Current studies on respiratory airway microbiota have predominantly focused on populations from the northern hemisphere, including the USA (26), Taiwan (16), China (36) and Portugal (30). However, some evidence indicates that the microbiota is affected by factors such as ethnicity, diet, and geographic zone (37, 38). Consequently, the relationships between certain bacteria and asthma in northern populations might not hold for other populations around the world. For instance, the Human Microbiome Project showed that bacterial taxa found in healthy US subjects are not universal, neither in the body sites studied nor in the subjects (39). In Ecuador, for example, members of the genus *Streptococcus* are detected in oropharyngeal samples in higher abundances than in Europe or the USA, although the implications of this are not yet fully understood (40). In part this has prompted the rise of microbiome centers with a regional component such the American Gut (41) and MetaHIT Consortium (42). Geographic variation is also true for environmental microbiomes as demonstrated for example by the MetaSUB Consortium (43). Studies related to asthma in Chile are limited to socioeconomic factors (44) and prevalence (6, 45), are clinically descriptive (46), and address treatments (47) related to the disease. Therefore, the diversity of the airway microbiota in healthy and asthmatic Chileans is still unknown.

In this study, we assessed whether the oral and nasal microbiota vary in composition, structure, and function between healthy and asthmatic children from Santiago de Chile. Moreover, we explored microbial interactions in both groups to identify relevant (keystone) taxa in the structure of each microbiota. Toward these goals, we characterized the nasal and oral microbiota of 152 subjects using 16S rRNA gene amplicon sequencing and compared their taxonomic and functional diversity.

## Materials and methods

### Study population and sample collection

All study participants were volunteers. Swab samples were collected at the Amador Neghme primary health center in Santiago, Chile. Written consent was obtained from parents or legal guardians of the volunteers before collecting samples. The study and consent documents were approved by the Ethics Committee of “Servicio de Salud Metropolitano Sur”.

Healthy and asthmatic children from the Santiago area were enrolled in the fall and winter of 2016, 2017 and 2018 to participate in this study. Asthmatics were diagnosed according to AUGE diagnostic clinical guidelines of the Ministry of Health, Chile for children under 15 years of age, which in turn are based on The Agency for Healthcare Research and Quality (48). In brief, the evaluation criteria are a clinical history of the disease, difficulty in breathing, spirometry evaluation with bronchodilator response, and increased forced expiratory volume (equal to or greater than 12% after receiving the application of 400 µg of salbutamol after 15 min). Patients who did not present these characteristics or presented any of the exclusion criteria (see Supplementary File S1) were not eligible for the study.

Swab samples were taken from both the nostrils and oral cavity of asthmatic patients and healthy subjects following and adapting the sample extraction protocol of the Human Microbiome Project (HMP) (49). Sterile swabs were rubbed against the walls of the mouth (∼4 cm^2^) or both nostrils for 20 to 40 s to ensure transfer of microorganisms to the TD1 buffer solution of the Ultra Clean Tissue and Cell kit (MoBio Laboratories). Swabs were immersed in 700 µl of TD1 buffer (UltraClean Cell and Tissue DNA Isolation Kit) and stored at −20°C until the DNA extraction procedure. Oral samples from individuals with “chronic dry mouth”, periodontal lesions, oral abscesses, or evidence of candidiasis were discarded. Similarly, only nasal samples from individuals vaccinated with a live attenuated influenza vaccine administered through the nose at least 28 days ago, no signs of inflammation, polyps or masses, and no infection of the nasal cavity and upper respiratory tract were considered. The healthy subjects enrolled in the study met the following criteria: not presenting any disorder or disease of the upper or lower respiratory tract, not having active antibiotic or antibiotic treatment for 2 months prior to the sampling and not presenting any exclusion criteria from the study (Supplementary File S1).

### DNA extraction and sequencing

Total DNA was extracted using the UltraClean Tissue & Cells DNA Isolation Kit (Cat No. 12334-S, MO BIO Laboratories, Inc.). Samples were homogenized on a horizontal Vortex Adapter (Catalog #13000-V1, MO BIO Laboratories, Inc.) following the manufacturer’s instructions. The concentration of DNA was quantified by a Qubit® 3.0 Fluorometer (Invitrogen), using a Qubit dsDNA HS Assay Kit (Cat N° Q32854). Each DNA sample was amplified for the V4 region of the 16S rRNA gene and libraries were prepared and sequenced using the Schloss MiSeq_WetLab_SOP protocol (50). Twenty nasal and oral samples were sequenced at The Microbial Systems Molecular Biology Laboratory (MSMBL) sequencing group (University of Michigan, Ann Arbor, MI, USA), while 160 samples (nasal and oral mucosa) were sequenced at The Environmental Sample Preparation and Sequencing Facility at Argonne National Laboratory (Lemont, IL, USA). The sequencing facilities used in this study used negative and positive controls in their protocols. Positive controls (Zymo microbiomics) were verified, though were not formally included in the analysis. After quality control and filtering 28 samples were discarded and 152 samples were analyzed. All sequence data was deposited in the NCBI under Bioproject accession number PRJNA446042. All R code and metadata are available in GitHub (https://github.com/ramostapiai/16s-Analysis).

### Microbiota analysis

Forward and reverse reads were trimmed at 150 bp to maintain quality over PHRED 25 and filtered using the following parameters: maxN = 0, maxEE = c(2,2), truncQ = 0, rm.phix = TRUE. Error rate learning, dereplication, and read merging were performed using default settings. Taxonomy was assigned using the Silva database for the16S rRNA gene (51) (version 132). A multiple sequence alignment was carried out using the R DECIPHER package (52) (version 2.7.3) and the phylogenetic tree was inferred using FastTree (53) (version 2.1.10). We created a phyloseq object for subsequent microbial analyses using the Phyloseq package (version 1.23.1). Sixteen-S rRNA–V4 amplicon sequence variants (ASV) in each sample were inferred using DADA2 version 1.18 (54). Following the author’s recommendations, we discarded samples of <1,000 reads, uncharacterized (NA) phylum-level taxa, ASVs with an average relative abundance <1 × ^−5^ and ASVs that were not observed more than 2 times in at least 10% of the samples. To avoid batch effects, the sequence files coming from each sequencing facility were processed separately using DADA2 under default parameters (54). We then combined the inferred ASVs and generated a single phyloseq object according to the author’s recommendations (55). We normalized our samples using the negative binomial distribution as recommended by McMurdie and Holmes (56) as implemented in the Bioconductor package DESeq2 (57).

Taxonomic alpha diversity (Chao1, ACE and Shannon) was estimated in R using the phyloseq function estimate_richness (Phyloseq version 1.23.1). Phylogenetic diversity was estimated with estimate_pd (btools package of R version 0.0.1). Statistical differences between groups were assessed using Linear Mixed Effects (LME) model as implemented in the lme4 R package (v1.1–21) (58). In our LME model we included alpha diversity indices and taxa (phyla and genera) abundances (response variables) and health status (healthy or asthmatic; predictors), while accounting for non-independence of subjects (random effects). We included random effects in our LME models since some participants were sampled twice during the study. We also considered the potential contribution of clinical characteristics of the cohort on the composition of the microbiota. Therefore, other covariables were also initially included in our LME analyses (sample collection date, use of drugs, age, condition of the host, and gender). We also included “sequencing facility” to further account for potential batch effects. To avoid redundancy, we did not include three variables (use of drugs, age, and gender) used to predict health status (healthy or asthmatic). We also tested LME models with random intercepts and random slopes and different orders of factors. Initial LME models including the variables listed above were compared using the function lmerTest, which performs automatic backward elimination of factors. ANOVA type III tests with Satterthwaite approximation for degrees of freedom were also carried out for hypothesis testing.

Beta diversity was estimated using weighted and unweighted Unifrac distances. Dissimilarity between samples was explored using Principal Coordinates Analysis (PCoA). Indices were compared using permutational multivariate analysis of variance (adonis2) as implemented in the vegan R package version 2.5–6 (59). Models were compared using the Akaike Index Criterion (60) and significance was determined through 10,000 permutations. We also included “sequencing facility” as a factor in the permutational analysis to account for potential batch effects (*R*^2^ < 0.02).

Community interactions among bacterial taxa were inferred using the network approach implemented in the SPIEC-EASI R package version 1.0.5 (SParse InversE Covariance Estimation for Ecological Association Inference) (61) under the neighborhood selection (mb) model. Keystone species at the ASV level (hub nodes) were calculated using the node degree and node centrality metrics (Degree >5 and Betweenness >200). The degree value of a node represents the number of edges connected to the node. Betweenness is also a measure of centrality of the nodes that make up the network. The betweenness value of a node is calculated as the total number of shortest paths from all nodes to all other nodes that pass through the node in question.

Microbial functional signatures (i.e., metabolic pathways) based on 16S rRNA gene sequences were predicted using Phylogenetic Investigation of Communities by Reconstruction of Unobserved States (PICRUSt2) version 2.2.0-b (62). Differentially represented pathways were calculated via the Welch’s *t*-test (63) (CI = 0.95) with Benjamini-Hochberg correction-FDR (64) as implemented in the STAMP software (65).

Visualizations of alpha and beta diversity indices, microbial relative abundances, and co-occurrence networks were carried out in RStudio (version 1.2.1335) and R (version 3.6.1).

## Results

### Microbiome composition and diversity

After filtering and quality control, 152 samples (69 oral and 83 nasal) were obtained (Table 1 and Supplementary Table S1), which included a total of 4,921,605 sequences (mean = 27,001, median = 23,430), ranging from 2,023 to 91,604 sequences per sample and 120 ASV.

**Table 1:** Relevant variables collected for this study.

	Asthmatic (63 samples)		Healthy (89 samples)	
	Nasal mucosa	Oral mucosa	Nasal mucosa	Oral mucosa
Male	16	13	29	23
Female	19	15	18	18
Age [average (median/SD)]	6.6 (6/4.6)	8 (6.5/5.2)	8.4 (6.0/5.4)	10.2 (13.0/5.7)
Bronchodilator use (%)	32 (91.4)	25 (89.3)	4 (8.3)	2 (4.9)
Antihistamine use (%)	0 (0)	0 (0)	2 (4.1)	1 (2.4)

The chi-square statistic is 44.8 and 49.7 for nasal and oral mucosa in Bronchodilator use, respectively (*P*-value <0.0001). The other variables do not present significant differences between asthmatic and healthy samples.

Nasal microbiomes included sequences corresponding to four dominant phyla (>1%): Firmicutes (41%), Proteobacteria (41.8%), Actinobacteria (15.5%) and Bacteroidetes (1.3%). These phyla comprise 7 dominant genera (>1%) genera: *Moraxella* (24.1%), *Dolosigranulum* (17.4%), *Corynebacterium_1* (13.9%), *Streptococcus* (10.8%), *Staphylococcus* (10.5%), *Haemophilus* (9.8%) and *Veillonella* (1%). On the other hand, the oral microbiome included sequences that corresponded to five dominant phyla (>1%): Firmicutes (53.2%), Proteobacteria (31.4%), Bacteroidetes (9%), Actinobacteria (3.1%) and Fusobacteria (3.1%). Those phyla comprised 7 dominant (>1%) genera: *Streptococcus* (35.7%), *Haemophilus* (16.5%), *Gemella* (7.4%), *Veillonella* (6.5%), *Neisseria* (5.5%), *Porphyromonas* (3.9%) and *Moraxella* (1.2%) (Table 2, Supplementary Figures S1, S2).

**Table 2:** Mean alpha-diversity indices, beta diversity indices, and mean relative abundance of dominant phyla and genera (>1%) of nasal and oral samples.

Alpha-diversity	Nasal mucosa				Oral mucosa			
	Asthmatic	Healthy	i (DF)	*P*(>*F*)	Asthmatic	Healthy	*F* (DF)	*P*(>F)
ACE	19.9	28.81	39.37 (48.0)	*P* < 0.01	54.7	49.4	0.4 (57.0)	ns
Shannon	1.1	1.55	13.4 (43.0)	*P* < 0.001	2.5	2.4	1.49 (60.1)	ns
Chao1	18.4	26.4	19.25 (60.0)	*P* < 0.001	54.2	49.1	0.39 (62.0)	ns
PD	3.3	3.99	9.78 (38.6)	*P* < 0.01	5.9	5.6	0.01 (62.0)	ns
Beta-diversity								
Jaccard	–	–	2.5 (1.0)	*P* < 0.001	–	–	1.1 (1.0)	ns
Bray-Curtis	–	–	3.37 (1.0)	*P* < 0.001	–	–	1.1 (1.0)	ns
Unifrac	–	–	3.2 (1.0)	*P* < 0.05	–	–	1.2 (1.0)	ns
Wunifrac	–	–	3.8 (1.0)	*P* < 0.01	–	–	0.3 (1.0)	ns
Phylum—% (SD)								
*Proteobacteria*	50.1 (32)	35.7 (32)	5.4 (51.5)	ns	30.1 (16)	32.3 (18)	2.8 (62.7)	ns
*Firmicutes*	36.8 (22)	44.1 (25)	6.8 (59.2)	ns	53.4 (17)	53.1 (19)	4.5 (55.8)	ns
*Actinobacteria*	12.7 (19)	17.5 (21)	0.5 (48.5)	ns	3.4 (3)	2.9 (2)	0.37 (69.0)	ns
*Bacteroidetes*	0.3 (0.6)	2 (5)	1.4 (67.2)	*P* < 0.05	9.2 (7)	8.8 (7)	65.7 (0.08)	ns
*Fusobacteria*	–	–	–	–	3.7 (3)	2.7 (2)	1.2 (64.3)	ns
Genus—% (SD)								
*Moraxella*	33.1 (30)	17.5 (28)	5.7 (62.7)	ns	2.9 (15)	0 (0.04)	1.8 (2,141.0)	ns
*Haemophilus*	14.3 (24)	6.5 (12)	4.6 (41.5)	ns	14.4 (9)	18 (13)	0.11 (69.0)	ns
*Dolosigranulum*	21.5 (20)	14.5 (0.1)	0.4 (59.0)	ns	–	–	–	–
*Streptococcus*	8.1 (10)	12.7 (16)	64.1 (4.5)	*P* < 0.01	34.4 (14)	36.5 (14)	3.1 (60.9)	ns
*Corynebacterium_1*	12.5 (19)	14.9 (20)	0.1 (50.1)	ns	–	–	–	–
*Veillonella*	0.5 (1)	1.4 (3)	2.7 (66.8)	ns	6.2 (6)	6.7 (6)	1.7 (60.9)	ns
*Staphylococcus*	6.3 (13)	13.6 (21)	3.7 (44.1)	ns	–	–	–	–
*Neisseria*	0.1 (0.3)	1.1 (1)	9.3 (65.3)	*P*< 0.001	5.4 (6)	5.6 (5)	0.18 (60.9)	ns
*Gemella*	–	–	–	–	8.4 (6)	6.7 (5)	0.11 (61.9)	ns
*Porphyromonas*	–	–	–	–	3.6 (4)	4.1 (5)	0.01 (65.8)	ns

Linear mixed-effects (LME) model results are shown for alpha-diversity indices and taxa abundances, while permutational multivariate analysis of variance (adonis) results are shown for beta-diversity indices. The significance of LME models was estimated using ANOVA of type III with Satterthwaite approximation for degrees of freedom. For each test, we report the relevant F statistic, degrees of freedom (DF), and significance [*P*(>*F*)].

**Figure 1:**
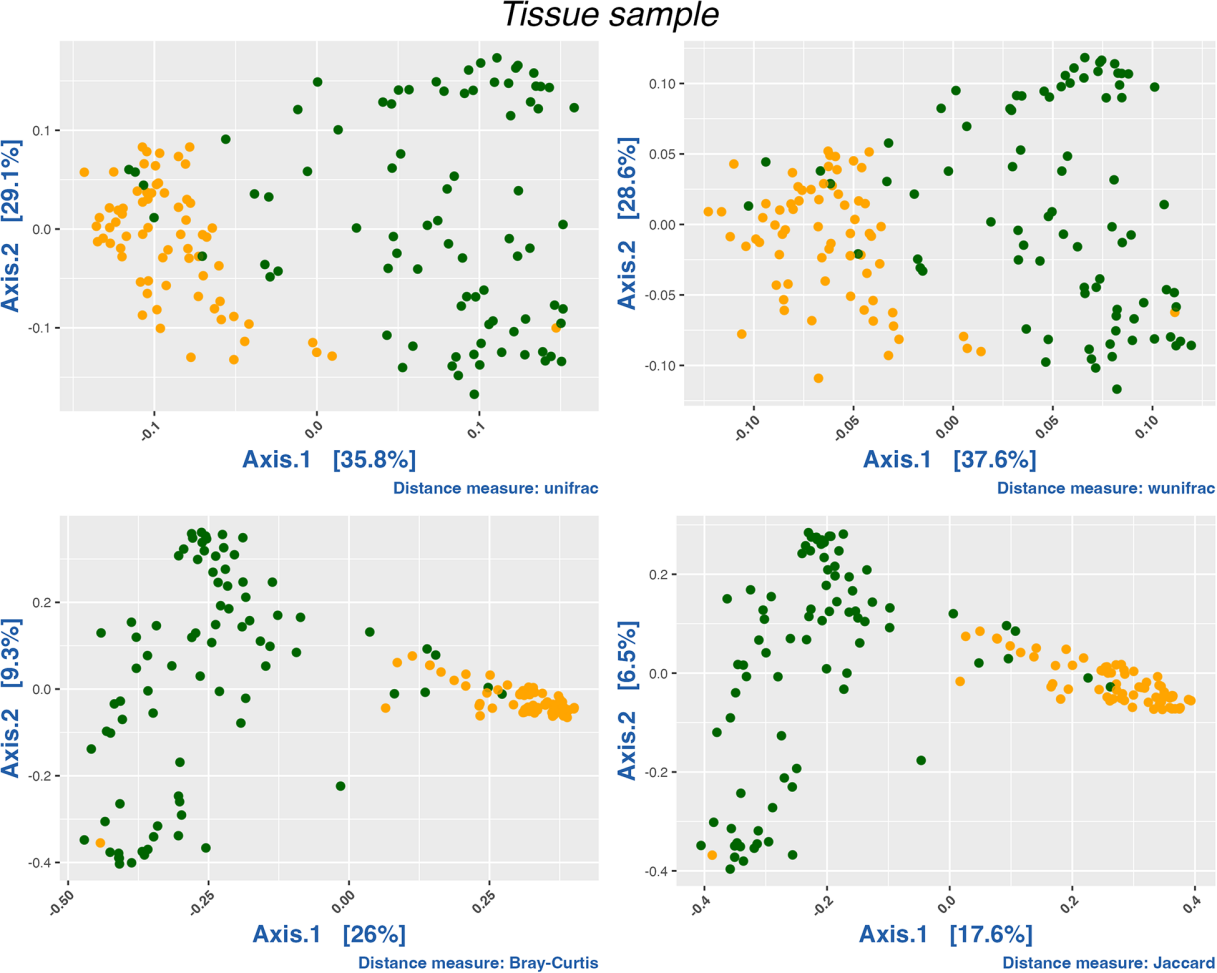
PCoA analysis of all samples by compartment. Oral samples in orange and nasal samples in green. We conducted a PERMANOVA test using distance matrices (Jaccard, Bray-Curtis, Unifrac and Wunifrac; *P* < 0.0001). R2 for Jaccard (0.14097), Bray-Curtis (0.21004), Unifrac (0.26966) and wUnifrac (0.20951).

The nasal and oral microbiomes differ greatly in species turnover as indicated by the PCoA of weighted Unifrac distances and confirmed by the Permutational Multivariate Analysis of Variance (PERMANOVA) (*P* = 9.999 × ^−5^, *R*^2^ = 0.26966). While the oral mucosa samples are similar to each other, the nasal microbiome exhibits a wide dispersion among samples, which indicates that its composition, phylogenetic, and taxonomic structures are more variable (Figure 1). Likewise, we did not find any clear patterns structuring oral microbiomes regarding their disease status, i.e., microbial communities coming from asthmatic patients did not seem to differ substantially in species turnover from healthy individuals (Supplementary Figure S3).

**Figure 2:**
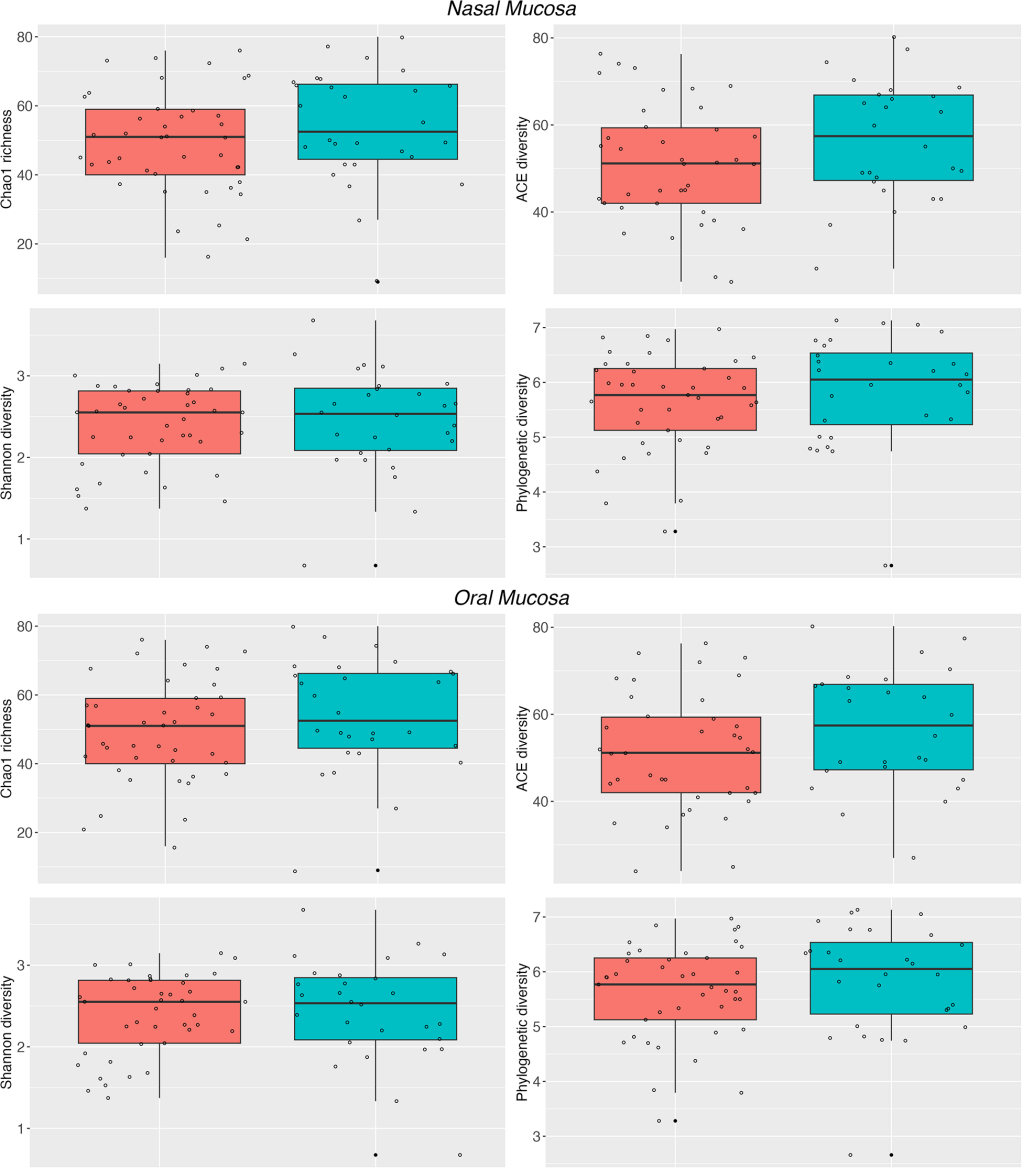
Alpha diversity of the nasal and oral microbiota. Nasal (top panel) and oral (bottom panel) microbiota from healthy subjects (cyan) and asthmatic patients (red). The Chao1, ACE, Shannon and PD indices show differences between the asthmatic and healthy nasal microbiota. See Table 2 for detailed statistics.

The microbiota of asthmatic and healthy children in both the nasal and oral compartments shared many bacterial genera, but differed in their relative proportions, especially in the nasal compartment. Our LME analyses of nasal samples from healthy and asthmatic subjects showed significant differences in the phylum Bacteroidetes (*P* = 0.03787), and the genera *Streptococcus* (*P* = 0.011443) and *Neisseria* (*P* = 0.0002554) (Table 2). Similarly, our LME analyses of the oral mucosa did not show differences in the abundances of the phyla or genera (Table 2). The oral microbiome did not show significant differences in alpha diversity between asthmatic and healthy subjects (Table 2 and Figure 2).

Conversely, the results in the nasal communities showed that the diversity between asthmatic and healthy subjects varies greatly (Permanova, Unifrac, wUnifrac, Bray-Curtis and Jaccard index; *P* > 0.01) (Table 2 and Supplementary Figure S4). In particular, nasal microbiomes showed significant differences in alpha diversity between the asthmatic and healthy subjects in indices Chao1 (*P* = 4.699 × ^−5^), ACE (*P* = 9.513 × ^−8^), Shannon (*P* = 0.0006668), and phylogenetic diversity (*P* = 0.0033340) (Table 2). Altogether, these results suggest that the microbiota is structured differently in the upper airways and that there is a significant difference between asthmatic and healthy subjects in the nasal mucosa. We did not observe significant differences in the diversity of the oral microbiota and results show no evidence of community differences between asthmatic and healthy subjects in the oral samples, except for a trend in the abundance of Firmicutes.

### Microbiome interactions

**Figure 3:**
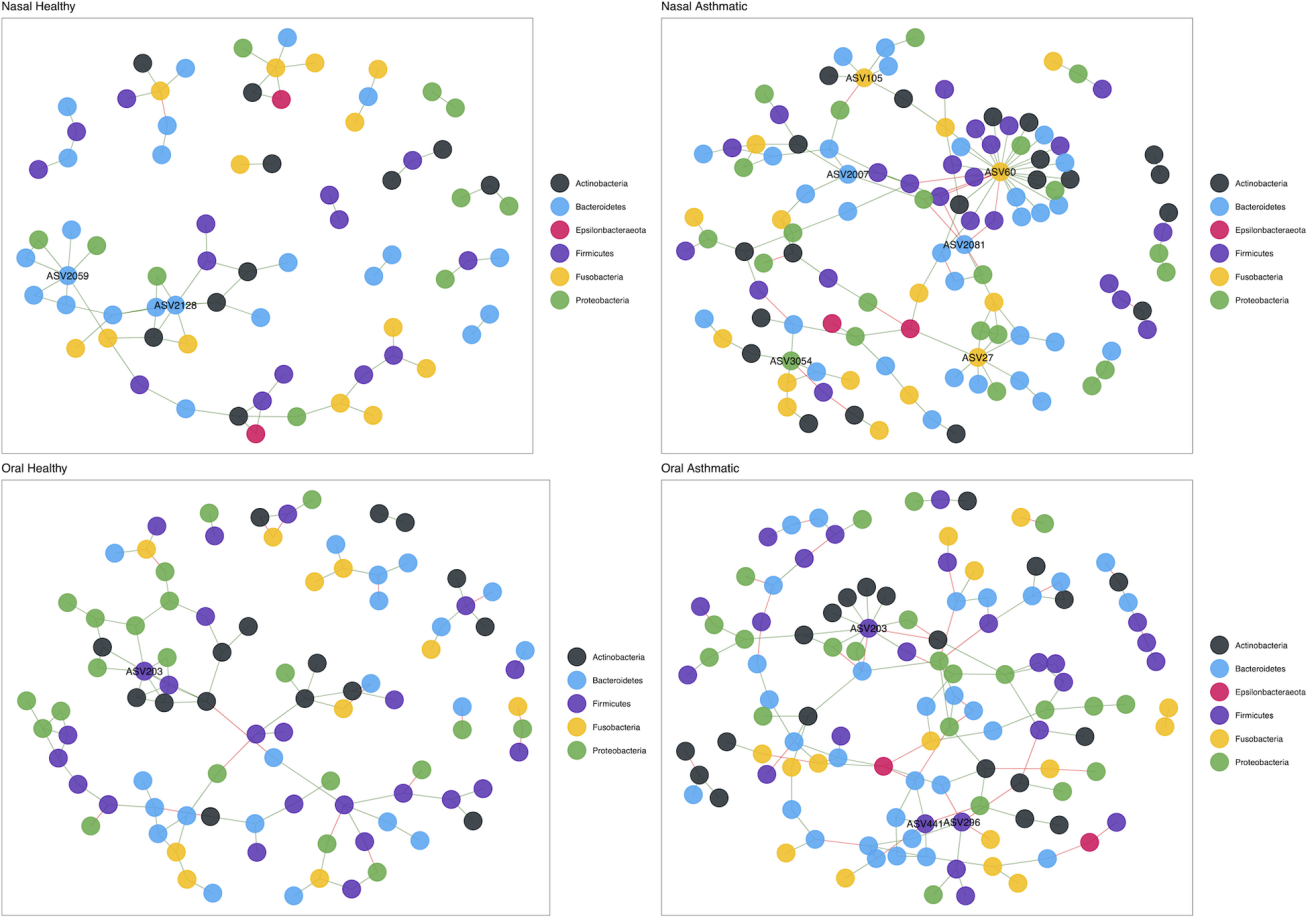
Structure of the nasal microbiota in health and disease. Upper panel shows nasal microbiota co-occurrence networks in healthy (left) and asthmatic (right) subjects. Bottom panel shows oral mucosa co-occurrence network in healthy (left) and asthmatic (right) subjects. Colors represent taxa at the phylum level.

To infer interactions between bacteria and identify keystone taxa (ASVs), we performed a co-occurrence network analysis. The nasal microbiome networks showed clear differences in topology and number of keystone taxa between asthmatic and healthy subjects (Figure 3). The oral microbiota was characterized by keystone ASVs of the genera *Leptotrichia* (ASV27: degree = 27 and betweenness = 726; ASV60: degree = 8 and betweenness = 2,250; ASV105: degree = 6 and betweenness = 491), *Porphyromonas* (ASV2007: degree = 5 and betweenness = 764), *Prevotella*_6 (ASV2081: degree = 5 and betweenness = 661), and *Kingella* (ASV3054: degree = 5 and betweenness = 985). In turn, the nasal microbiome network of healthy children included keystone ASVs of the genera *Prevotella*_2 (ASV2059: degree = 7 and betweenness = 160) and *Prevotella*_7 (ASV2128: degree = 7 and betweenness = 146). The nasal microbiome networks also showed differences in topology between asthmatic and healthy subjects, but the keystone taxa did not vary much; a ASV of the genus *Streptococcus* (ASV203: degree = 11 and betweenness = 1,085) was observed in both healthy and asthmatic children, while two ASVs of the genera *Granulicatella* (ASV296: degree = 6 and betweenness = 777) and *Veillonella* (ASV441: degree = 6 and betweenness = 900) were found only in the asthmatic subjects.

### Microbiome functional profiles

**Figure 4:**
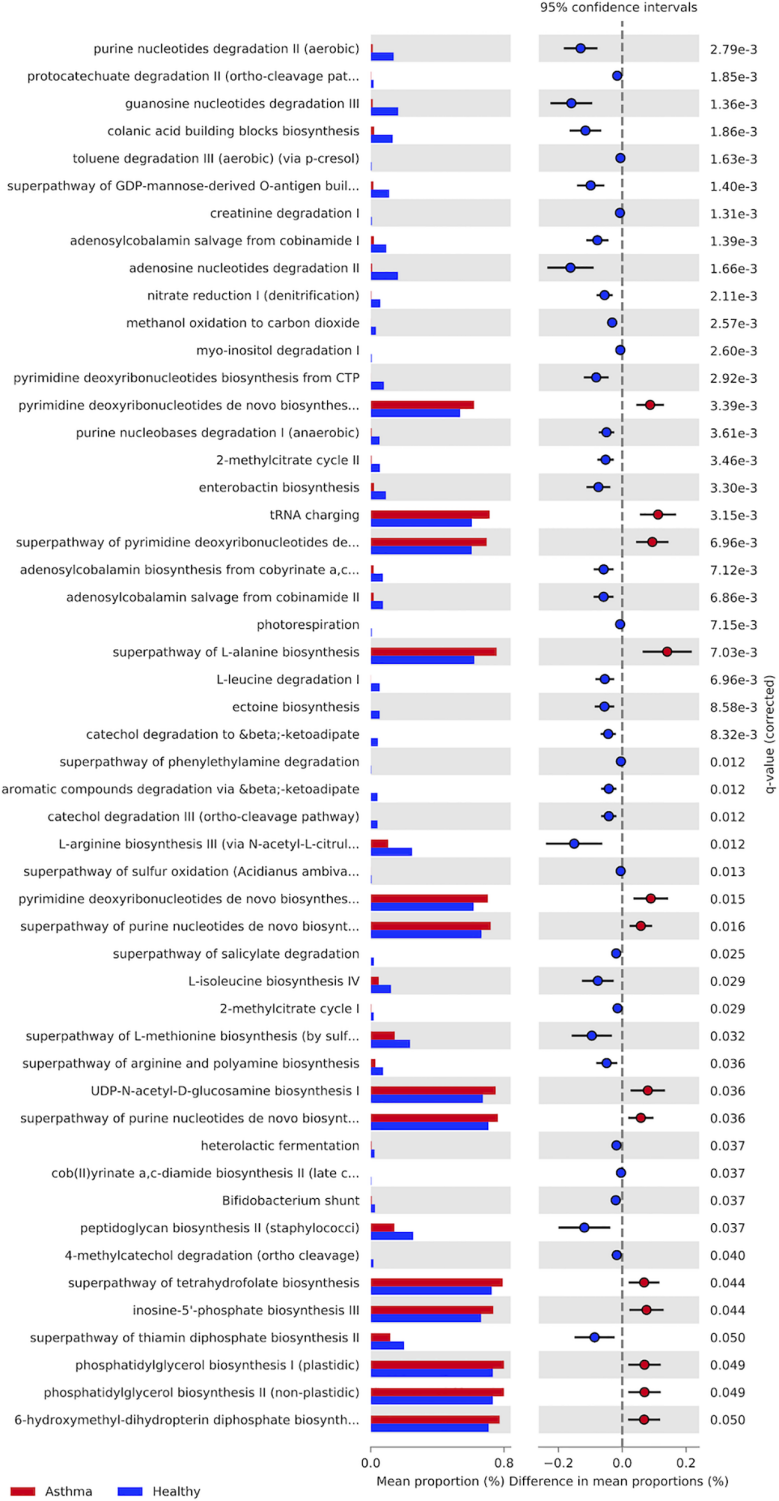
Metabolic pathways present in the asthmatics and healthy subjects’ nasal microbiota. Asthmatic subjects in red and healthy subjects in blue. Last column shows *P*-value (*P* > 0.05).

We found significant differences in the relative abundances of metabolic pathways between nasal microbiomes of asthmatic and healthy subjects (Figure 4 and Supplementary Table S2). We identified a total of 51 metabolic pathways, of which a total of 38 pathways were significantly underrepresented in asthmatics; these pathways are related to the degradation and synthesis of amino acids, nucleotides, and carbon sources. Only 13 pathways were significantly overrepresented in asthmatics, including tRNA charging pathway, L-alanine biosynthesis, pyrimidine deoxyribonucleotides biosynthesis/degradation and purine nucleotides biosynthesis. With respect to the oral mucosa, we did not detect any significant differences in metabolic pathways between healthy and asthmatic subjects.

## Discussion

Asthma is a condition that imparts large economic burdens to individuals and society. While previous studies indicate that multiple factors are related to the onset and development of asthma, emerging evidence suggest that asthma is intimately linked to alterations of the upper airway microbiota. In this cross-sectional study, we applied 16S rRNA gene sequencing to a cohort of children and adolescents from Santiago de Chile. We identified distinct taxonomic and functional profiles, as well as significant differences in the structure of the microbiota and key bacterial taxa.

### Diversity and composition of the nasal and oral microbiotas in asthmatic and health subjects

Our results show differences in diversity and composition in the nasal microbiota of asthmatic subjects compared to healthy subjects (Table 2). We detected a decrease in alpha diversity in the nasal microbiota of asthmatic individuals (Figure 1). Decreased microbial diversity has also been observed in other human diseases, e.g., intestinal failure (66), diarrheagenic infection (67) or intestinal inflammation (68), including some related to the airways, such as rhinitis, bronchiolitis, and cystic fibrosis (18, 30, 69, 70). Our results also agree with previous studies in other populations that show that microbial communities of asthmatics are less diverse than those of healthy individuals (25, 26, 71, 72), which we interpret as a result of the overdominance of some taxonomic groups, and the resulting changes in community structure and microbial interactions over the whole of the community.

Regarding the diversity of the oral microbiota, our analyses did not show significant differences in any diversity index evaluated, indicating that there is no significant variation in the oral microbiota among asthmatic subjects compared to healthy individuals. These results suggest that the oral microbiota seems to be resistant in this particular cohort. We chose the oral mucosa as the relevant sample to study because relationships between the oral microbiota and its involvement in respiratory diseases or complications are unknown. Associations between microbes in the oral cavity and respiratory diseases have been identified in other studies. In pneumonia, for example, respiratory pathogens such as *Haemophilus influenzae* and *Klebsiella pneumoniae* are readily detected (73); *Staphylococcus aureus* (74) and SARS-CoV-2 have also been detected recently (75). In addition, other studies have shown that subjects with COPD have poor oral health and, in consequence, alterations in the oral microbiota (76, 77). Also, recent studies show significant changes in composition and structure of the microbial communities in asthmatics, rhinitis patients, and controls (78). Although certain microorganisms have been found that could be associated with allergies or asthma, such as *Gemella* or *Lactobacillus* (79); *Veillonella*, and *Streptococcus* (80); and *Aggregatibacter* (81), they have not shown dysbiosis in the oral microbiota during asthma.

Previous studies have also shown that the composition of the microbiota varies across human populations (37, 39, 82). Likewise, the oral and nasal microbiota, either in health or disease, have been shown to vary across populations (83–86). In this study, we found that the nasal microbiota of asthmatic subjects has a high relative abundance of *Moraxella* and *Haemophilus* (Table 2). These results partially agree with previous studies on cohorts from different countries. For instance, in the USA the most abundant taxa in asthmatic subjects are *Moraxella, Streptococcus* and *Haemophilus* (24–26). In one population in Europe, the more common taxa are *Streptococcus*, *Veillonella*, *Haemophilus*, *Prevotella*, and *Rothia* (87), while in Portugal high abundant taxa in asthmatic subjects were *Dolosigranulum, Haemophilus, Lawsonella,* Moraxella, and *Neisseria* (30). Therefore, the same microorganisms have been detected, however, they differ in their relative abundances, which suggests that this depends on each population under study. The overabundant taxa possibly involved in dysbiosis (*Moraxella* and *Haemophilus*) are shared with other respiratory diseases such as bronchitis (28) or rhinitis (88), which are chronic diseases of the respiratory tract, producing inflammation and dysfunction of the nasal mucosa (89, 90).

In terms of composition, our findings show large variability across subjects with and without asthma (Table 2). However, the genera *Moraxella*, *Dolosigranulum*, *Haemophilus*, *Corynebacterium_1*, *Streptococcus* and *Staphylococcus* dominated most of the samples of the asthmatic nasal mucosa. In studies related to asthma in children, the same consortium of bacteria (*Moraxella*, *Streptococcus*, *Haemophilus*, *Corynebacterium_1*, *Dolosigranulum* and *Staphylococcus*) has been reported in other tissues and samples, frequently associated with respiratory tract diseases (17, 25–28, 91, 92). In the same way, the nasal mucosa of healthy children is characterized by the presence of the same genera listed above, all previously reported in the literature (26, 71, 88).

For the oral microbiota in both types of individuals, the most prevalent and abundant genera were *Streptococcus*, *Haemophilus*, *Gemella*, *Vellionella*, *Neisseria*, and *Porphyromonas*. Only the phylum Firmicutes varied significantly in abundance between healthy and asthmatic subjects. This could be, at least in part, because the oral microbiota is associated with other human diseases such as periodontitis, refractory periodontal disease, caries, and odontogenic infection (93). Other works associate early changes in the oral microbiota with maturation of the immune system and the development of allergies or asthma (79). However, a study on an African American cohort showed significant changes in the composition of the microbiota in saliva samples (oral cavity) with an increase in the genera *Streptococcus* and *Veillonella* in asthmatic subjects (80).

The characterization of the oral microbiota is consistent with other studies. For instance, studies in adults using oropharynx and oral wash samples and 454 pyrosequencing detected a high relative abundance of Streptococcaceae, Veillionellaceae, Fusobacteriaceae and Neisseriaceae (94)*.* Hilty and collaborators in 2010 reported the same phyla and genera in oropharynx samples, one of the first studies related to asthma and microbiota (20). Other studies have found similar results than those reported here, e.g., high abundances of *Streptococcus*, *Prevotella,* and *Veillonella*, which comprise 70% of the oral microbiota (95). However, more studies of the oral microbiota in children or adolescents are required, since recent studies have shown that the microbiota of children is more diverse than that of adults; however, the predominant microorganisms of the oral microbiota have similar abundances (96).

Furthermore, in our study we have observed limited variability of the oral microbiota between subjects, as shown by alpha and beta indices (Figures 1, 2). In contrast, other studies of different diseases or conditions, like children with obesity (97), have revealed changes in the diversity of the microbiota compared to children with normal weight, or changes in microbial diversity associated with gingival bleeding in children (98). In our cohort, we did not detect any evidence of change or alteration in composition or difference in diversity in the oral microbiota. We expect that a new analysis using a greater number of individuals may increase the power to detect variation in this compartment.

Finally, in oral samples, our results only showed a trend between healthy and asthmatic members of the Firmicutes phylum. This is not analogous to what has been suggested by other studies on asthma in children and young people carried out in a different cohort (African Americans) (80). This result reinforces the importance of local studies; cross-sectional studies in addition to longitudinal studies in different populations will be decisive in providing information linking oral microbiota dysbiosis with asthma.

### Nasal microbial function varies between asthmatic and healthy subjects

We detected differences in the representation of metabolic pathways on nasal mucosa in asthmatic subjects, e.g., in metabolic pathways related to nucleotide synthesis such as pyrimidine biosynthesis and purine biosynthesis, and pathways related to amino acids, metabolism and transport. These results suggest that the functional potential of the nasal microbiota could be subject to an imbalance as a result of microbial differences in composition between asthmatic and healthy subjects. Previous studies have shown similar results in unrelated populations (30, 99–103). Studies focused on other respiratory illnesses, such as cystic fibrosis have also shown major changes in the representation of metabolic pathways, e.g., enrichment in degradation of aminobenzoate, geraniol, lysine, benzoate, valine leucine and isoleucine; metabolism of beta-alanine, propanoate, tryptophan, butanoate and fatty acid (70).

Like asthma, rhinitis is a disorder in which immunoglobulin E (IgE) and Th2 lymphocytes mediate responses to a small numbers of allergens (104–106). Some studies estimate that 38% of subjects with asthma have rhinitis and that both conditions can coexist in the same patient (106). Metabolomic studies in allergic rhinitis show that deoxyuridine and inosine compounds (pyrimidine metabolism and purine metabolism, respectively), are mostly present in subjects with this disease (107). These same pathways are also overrepresented in asthmatic subjects (Figure 4). In addition, studies of allergic rhinitis with other cohorts show that the same metabolic pathways are increased in cases compared to controls (30, 108).

The alanine synthesis pathway also shows an overrepresentation in asthmatic subjects. Amino acids such as lysine, histidine, and tyrosine, among others, have been studied and are related to IgE sensitivity and response in mild and severe asthma (109). In the case of cystic fibrosis, changes in carbon sources are correlated with a dysbiosis in the microbiota, which might reflect changes in energy requirements in metabolizing carbohydrates (70). Similarly, pyrimidine and purine nucleoside triphosphates serve as precursors of DNA and RNA (110), which also suggest that an enrichment in these pathways is related to changing energy requirements. We speculate that the microbiota in asthma is more energy demanding than in the healthy microbiota, possibly due to challenges imposed by the host or by members of the microbiota.

These results suggest that in the studied cohort, metabolites from members of the microbiota could be related to inflammation in the upper pathways. Further studies using metabolomics or metatranscriptomics will be needed to identify which metabolites or active metabolic pathways are up- or down-regulated in asthmatic subjects. For example, certain compounds can modulate the microbiota and its metabolic activity (111) or how certain taxa contribute to asthma (112) or other diseases (COPD). Different omics approaches are being integrated to establish connections between microbiome-metabolome and the host (113).

### Bacterial interactions in the nasal and oral microbiotas of asthmatic and healthy subjects

We found four keystone species in the nasal microbiota of asthmatic subjects, i.e., *Leptotrichia*, *Porphyromonas, Prevotella*_6, and *Kingella*. These results suggest that differences in microbial diversity and composition may cause a restructuring of the microbial interactions in the nose and mouth, so different taxa may adopt key roles in the microbiota. Other studies addressing microbiota structure in asthma have also reported keystone species. In a study from a population in the USA that used a metatranscriptomic approach, researchers reported *Moraxella*, *Alloiococcus*, and *Corynebacterium* as keystone species (27), none of which were detected in our dataset. A study of a population from northern Portugal (30) using more than 300 samples also examined community structure using networks and reported that subjects with allergic rhinitis with and without asthma have more complex networks with more connected nodes, which is in accordance with what we report in this study, where the networks of asthmatic subjects have more connections and are more complex (Figure 4). In addition, the key taxa identified in the Portuguese cohort such as *Leptotrichia* and *Veillonela* were also detected in this study, though the Chilean population also identified the genus *Prevotella* as keystone, which was absent in the Portuguese population. This suggests that asthma has key and shared mechanisms at the microbiota level and that the diversity of the microbiota is a key point to consider in future studies. However, other studies in asthma have also revealed significant differences in co-occurrence networks when studied fungal and bacterial composition between endotypes of asthma (114) and asthmatic vs. controls (115, 116).

Overall, these results are consistent with the available literature, which suggests that *Prevotella* is a commensal bacterial genus, but in some cases exhibits pathobiont properties (117). *Prevotella* abundance has been reported to be reduced in subjects with COPD and with asthma (20). The genus *Prevotella* is associated with establishing tolerance in the respiratory airways as symbiotic bacteria and could partially reduce *Haemophilus*-induced IL-12 production by dendritic cells (118). In our case, we see *Prevotella* replaced by other key taxa, which could suggest that inflammation caused by changes in composition and the overabundance of certain taxa displaces its participation (119). This likely reflects different symbiotic interactions between pathogenic and commensal bacteria in the nose as seen in other respiratory diseases (120, 121). Nonetheless, microbial taxa co-occurrences shown here using 16S rRNA data need to be confirmed using more powerful shotgun metagenomic and RNASeq technologies (122).

Our findings are also in agreement with other studies that document that less abundant taxa may have a high degree of connectivity (123), as is the case of *Prevotella_2* and *Prevotella_7* in healthy nasal mucosa samples and *Leptotrichia*, *Porphyromonas*, *Prevotella_6*, and *Kingella* in the asthmatic nasal mucosa, which are identified as key nodes (i.e., keystone taxa). Interactions within communities are the fundamental support for their development and maintenance. Non-asthma studies using interaction networks within the microbiota have established that rare taxa play a central role in communities, for example, the genus *Symbiodinium* which plays a central role in coral robustness (124), or rare and low abundance taxa that contribute significantly to plant rhizospheres (125). This suggests that a particular node may play an irreplaceable role within the community by maintaining key interactions or relationships for its structure, regardless of their abundance.

The *Streptococcus* genus, one of the main culprits in respiratory tract diseases like pharyngitis and pneumonia (126) show a significant difference between healthy and asthmatic subjects (Table 2). However, we have not detected a high degree of connectivity or centrality within the network of this particular genus. Studies carried out in US American populations involving the use of metatranscriptomics have depicted *Streptococcus* as a hub that is negatively associated with all other members of the microbiota (27). However, traditional correlation analysis of microbial population amplicon data is likely to produce poor results (127). Studies based on Pearson’s correlation, as is the case of the study by Chun et al., do not account for independence between samples and, being purely compositional, are biased by the fact that, since they must sum to 1, the fractions are not independent and tend to be negatively correlated, regardless of the true correlation between the underlying absolute abundances (128). Therefore, estimates of correlations often reflect the nature of the composition of the data and are not indicative of underlying biological processes (129, 130). Recent methods that take into account the limitations of the techniques used and analyze the samples more independently generate a better representation of the microbial network (127, 131, 132). Our results do not consider the *Streptococcus* genus as a key node within the microbiota, which may be due to the variability of the microbiota across subjects. Future studies should estimate co-occurrence networks in the microbiota throughout the development of the disease. A study of disease dynamics and progression from a health to a disease would provide key information to assess how the interactions are modulated from a healthy microbiota to an imbalanced one (i.e., dysbiosis) (133).

In summary, this cross-sectional study characterizes for the first time the microbiota of the upper respiratory tract of Chilean children with and without asthma. We found diversity, compositional, and structural differences between asthmatic and healthy children in this particular cohort. The detected bacterial phyla and genera with differential abundance from those described in other cohorts of children with asthma. This reinforces the importance of comparative studies of the microbiome across human populations to discover differences and similarities and make more informed decisions about public health interventions.

## Data Availability

The datasets presented in this study can be found in online repositories. All sequence data was deposited in the NCBI under Bioproject accession number PRJNA446042. All R code and metadata are available in GitHub (https://github.com/ramostapiai/16s-Analysis).
