# Supplementary material for: Characterization of the upper respiratory tract microbiota in Chilean asthmatic children reveals compositional, functional, and structural differences
Source: Front Allergy. 2023 Jul 28;4:1223306. doi: 10.3389/falgy.2023.1223306 (PMC10419220; doi:10.3389/falgy.2023.1223306)
Supplement: Supplementary file 1 [file Datasheet1.docx]

**Subject Inclusion Criteria**

Asthmatic children who attend medical control consultations at the medical center and who participate will be eligible if they meet the following criteria:

- Diagnosis of asthma according to the AUGE Clinic guide (moderate and severe bronchial asthma in children under 15 years of age)
- No other diagnosis of respiratory disease
- Age between 1 - 16 years

**Patient exclusion criteria**

- Body mass index between less than 18 or more than 35.
- Vital signs outside the acceptable range, ie > 160/100, oral temperature greater than 37.7 C and pulse greater than 100.
- Patients who have used any of the following drugs in the last 6 months are excluded: Systemic, antiviral or anti-fungal/anti-parasitic antibiotics, whether intravenous, oral, or intramuscular.
- Not to take the sample if the patient has "chronic dry mouth", periodontal lesions of more than 4 mm, oral abscesses, evidence of precancerous lesions, evidence of candidiasis
- Use of oral, intravenous, intramuscular, nasal.
- Non-use of antibiotics for at least 1 month before sampling.
- Treatment with cytokines or Methotrexate or any cytotoxic or immunosuppressive drug.
- Large commercial doses of probiotics that are greater than or equal to 10^8 colony-forming units. These include capsules, tablets, chewing gum. Ordinary components of the diet such as fermented milk or yogurt are excluded.
- Have received the nasal influenza vaccine within 28 days prior to sampling.
- Use of topical antibiotics or steroids during the 7 days prior to sample collection.
- Some type of acute illness at the time of registration as a study participant.
- Any type of chronic disease except asthma in case of cases.
- History of cancer.
- Major changes in the diet, such as removal of some food group during the month prior to sampling.
- Recent history of alcohol use, defined as 5 or more servings of more than 44 mL per day.
- Positive test for HIV, HBV or HCV.
- Any confirmed or suspected condition or state of immunodeficiency or immunosuppression.
- History of psoriasis or eczema.
- For female patients, vaginal rings as contraceptives.
