# Supplementary figures and images for: Characterization of the upper respiratory tract microbiota in Chilean asthmatic children reveals compositional, functional, and structural differences
Source: Front Allergy. 2023 Jul 28;4:1223306. doi: 10.3389/falgy.2023.1223306 (PMC10419220; doi:10.3389/falgy.2023.1223306)

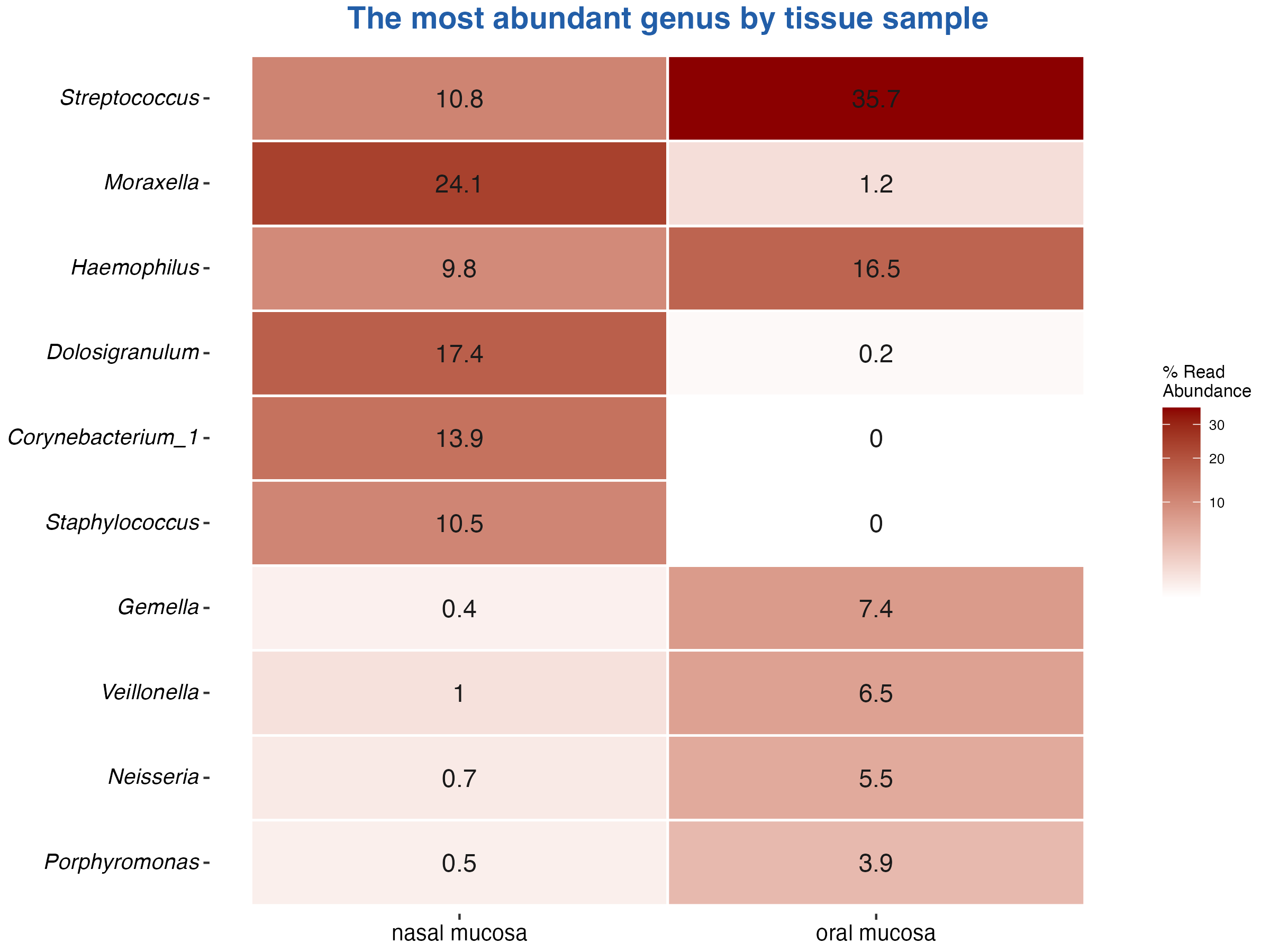

Supplement: Supplementary file 4 [file Image1.tiff]

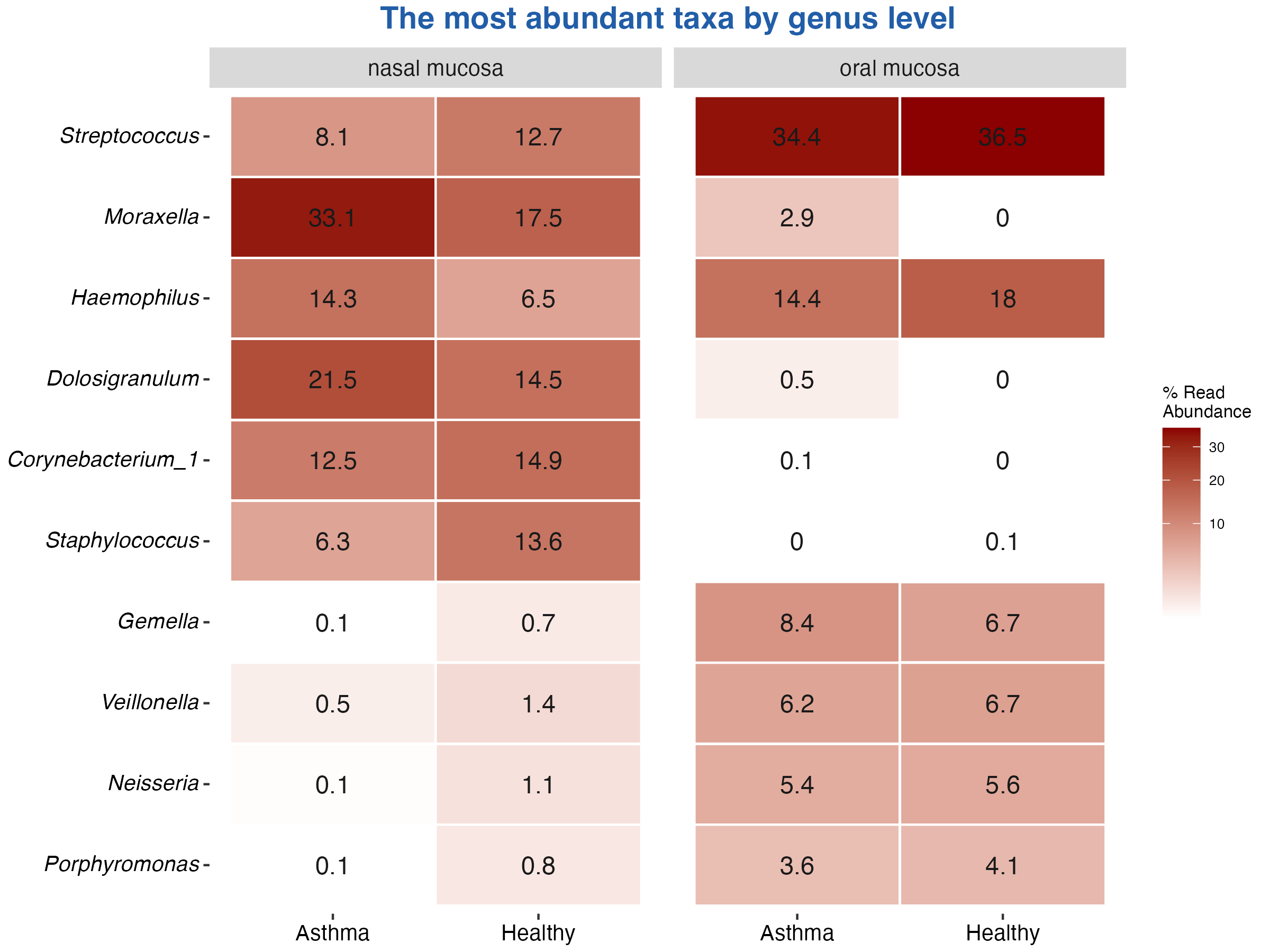

Supplement: Supplementary file 5 [file Image2.tiff]

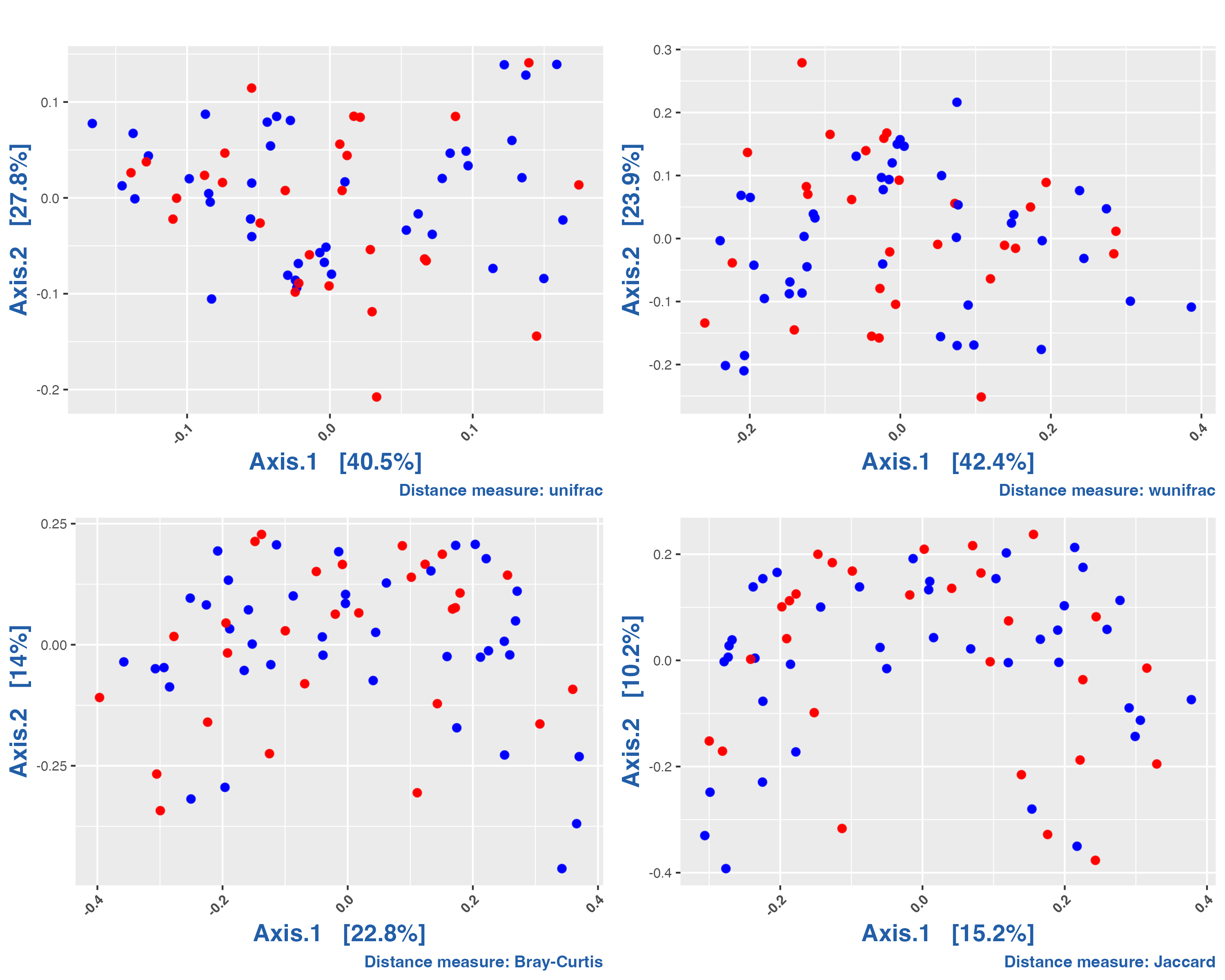

Supplement: Supplementary file 6 [file Image3.tiff]

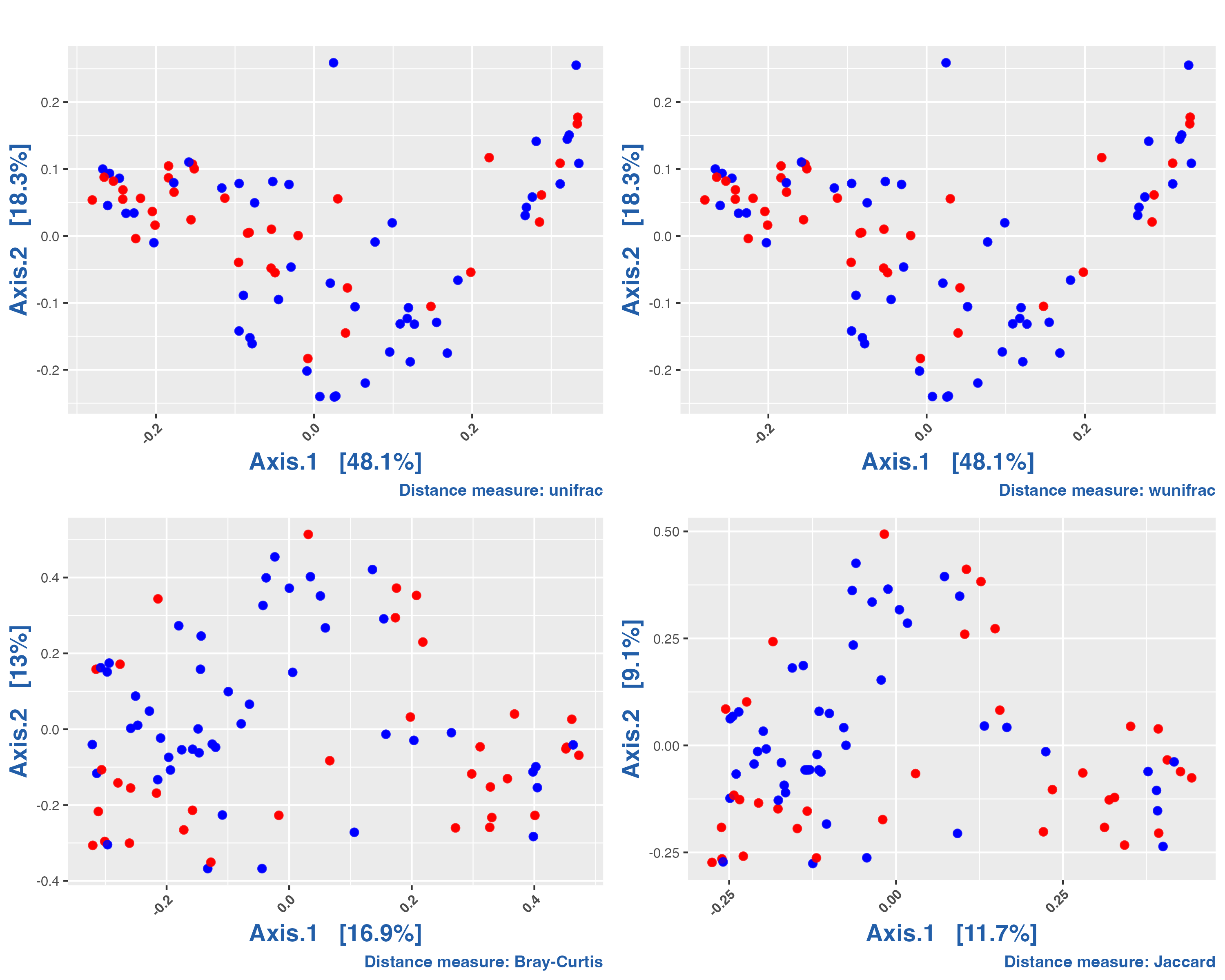

Supplement: Supplementary file 7 [file Image4.tiff]
